# Composted Green Waste as a Substitute for Peat in Growth Media: Effects on Growth and Nutrition of *Calathea insignis*

**DOI:** 10.1371/journal.pone.0078121

**Published:** 2013-10-29

**Authors:** Lu Zhang, Xiangyang Sun, Yun Tian, Xiaoqiang Gong

**Affiliations:** 1 College of Forestry, Beijing Forestry University, Beijing, P.R. China; 2 Institute of Desertification Studies, Chinese Academy of Forestry, Beijing, P.R. China; United States Department of Agriculture, United States of America

## Abstract

Peat mined from endangered wetland ecosystems is generally used as a component in soilless potting media in horticulture but is a costly and non-renewable natural resource. The objective of this work was to study the feasibility of replacing peat with different percentages (0, 10, 30, 50, 70, 90, and 100%) of composted green waste (CGW) as growth media for the production of the ornamental plant *Calathea insignis*. Compared with 100% peat media, media containing CGW had improved physical and chemical characteristics to achieve the acceptable ranges. Moreover, CGW addition had increased the stability (i.e., reduced the decomposition rates) of growth media mixtures, as indicated by comparison of particle-size distribution at the start and end of a 7-month greenhouse experiment. Addition of CGW also supported increased plant growth (biomass production, root morphology, nutrient contents, and photosynthetic pigment contents). The physical and chemical characteristics of growth media and plant growth were best with a medium containing 70% CGW and were better in a medium with 100% CGW than in one with 100% peat media. These results indicate that CGW is a viable alternative to peat for the cultivation of *Calathea insignis*.

## Introduction

Peat has long been used as a component in soilless potting substrates because of its homogeneous and favorable agronomic characteristics [1]. In recent decades, however, peat has been generally imported from Northern and Central Europe, and because it is a non-renewable resource, its price has been increasing [2], [3]. There has also been increasing environmental concerns that peat mining damages wetland ecosystems [4]–[6]. For these reasons, a substitute for peat as a potting substrate is needed.

Several possible substitutes have been recently described, and these include humus-like, composted organic waste [7]–[9]. In general, composted organic waste is increasingly being used as a substitute for or in combination with peat by the horticultural industry [10]. Research on the development of composted organic waste as a substitute for peat has been conducted with composts made with corn cobs, mushrooms, sewage sludge, municipal solid waste, and agro-industrial waste [3], [4], [10]–[12]. Composts used as components of potting substrates could be superior to peat because peat has poor nutrient availability, excessive water content, and suboptimum porosity [4], [5], [13]. On the other hand, the percentage of compost used must be carefully determined to avoid negative effects on plant growth. For example, high soluble salt contents of composts made with agriculture waste could limit their potential use in plant propagation [12].

With the development of urban greening, the quantity of green waste (i.e., fallen leaves and branch cuttings) has greatly increased in China and other countries. In China, most green waste is incinerated or deposited in landfills, but composting represents an attractive alternative from the standpoint of both environmental protection and economic development [8], [14]. Recycling of green waste through composting could not only reduce environmental problems caused by landfills and incineration but also decrease the cost of green waste disposal [15].

The current study concerns the use of composted green waste (CGW) produced by a two-stage composting process as a potting substrate for *Calathea insignis* [16]. *Calathea insignis*, which belongs to the Marantaceae family, is a perennial evergreen herb that is native to Brazil and other countries of tropical America. It is a very popular ornamental plant in China, where it is used indoors and is highly valued for its beautiful foliage. It produces leaves with dark green pinnate markings on the upper surface and a dark, purplish red lower surface. *Calathea insignis* requires acidic humic soil or peat. More specifically, it requires a growth medium that has high porosity and fertility, that is well-drained while retaining sufficient water, and that is also rich in nutrients. Few studies concerning the cultivation of *Calathea insignis* in peat substitutes have been performed.

The objectives of the present work were to study the physical and chemical characteristics of potting media created with a commercial peat (P) and different percentages of CGW, and to investigate the effects of the growth media mixtures on the growth parameters and nutrient contents of *Calathea insignis*.

## Materials and Methods

The performance of CGW as a potential peat substitute for the production of the ornamental plant *Calathea insignis* was investigated in a greenhouse at a privately owned nursery (the Shunyi District Nursery of the Beijing Green Garden Group) in Beijing, P.R. China from 31 March 2012 to 31 October 2012. The owner of the Shunyi District Nursery of the Beijing Green Garden Group (the Nursery Factory Director, Haiquan Wang) gave our research team permissions to conduct our studies in the nursery. That is, all necessary permits were obtained for the described studies, which complied with all relevant regulations. We also confirmed that the studies did not involve endangered or protected species.

### Growth Media Preparation

The growth media were prepared by mixing CGW and P, which was Pindstrup peat imported from Denmark, at different ratios. Seven CGW:P growth media mixtures (on a volume basis) were prepared: T1 (CGW:P = 0), T2 (CGW:P = 0.1), T3 (CGW:P = 0.3), T4 (CGW:P = 0.5), T5 (CGW:P = 0.7), T6 (CGW:P = 0.9), and T7 (CGW:P = 1). The mature compost used in this experiment was produced by a two-stage composting method (with primary and secondary fermentations) and with addition of fermentation additives (brown sugar and calcium superphosphate) as described by Zhang et al. [16]. The compost was made in a pilot plant using green waste (mostly fallen leaves and branch cuttings) collected in Beijing, P.R. China in the spring of 2011 during greening maintenance. The first stage of the composting process occurred in digester cells with non-covered, cement containers. When the fermentation temperature dropped to 45–55°C on day 6, the primary fermentation was considered complete. Then, composts from the digester cells were removed and arranged in windrows (2 m long, 1.5 m wide, and 1 m high) on a covered cement slab where the secondary fermentation occurred. The second stage of the composting process required addition of fermentation additives every 6 days beginning on day 6 (when the windrows were formed and the secondary fermentation began) and was considered complete when the fermentation temperature was stable and similar to that of the surrounding atmosphere. The production of mature compost requires only 30 days with the two-stage composting method but requires 90–270 days with the traditional composting process [16], [17]. Furthermore, the highest fermentation temperature is generally attained only once in traditional composting but is attained twice in the two-stage composting process, which reduces the probability that harmful organisms survive and which increases compost stability. The compost used in the current study was mature according to its carbon to nitrogen ratio (C/N ratio), which was less than 12, and according to its T value (T value = the final C/N ratio/the initial C/N ratio), which was less than 0.70 [16], [18].

### Greenhouse Experiment

The performance of CGW as a potential peat substitute was investigated in a greenhouse at a privately owned nursery (the Shunyi District Nursery of the Beijing Green Garden Group) in Beijing, P.R. China from 31 March 2012 to 31 October 2012. The greenhouse temperature was 20–25°C during the day and 18–20°C at night, with 65–75% relative humidity and 7,000 l× light intensity.

The experiment used 2000 cm^3^ plastic pots with holes in the bottom. As described by Jayasinghe, the air-dried growth media mixtures (media T1–T7) were blended mechanically and then added to each pot (leaving a distance of 1 cm from the top of the pots) without compaction in March 2012 [4]. Each of the seven media was represented by 30 replicate pots. The 210 pots were randomly placed on the shelves in the greenhouse, saturated with tap water (water hardness ≤10 d, pH between 6.5 and 8.5, and NaCl content <2 mmol/L), and then kept for 48 h so that they would drain to their respective field capacities. *Calathea insignis* seedlings were produced by tissue culture. When the plants were about 15 cm tall and had a fresh shoot weight of about 42 g and a fresh root weight of about 15 g, they were transplanted into the pots (one plant per pot). The plants were irrigated as needed, and the volume and timing of irrigation were identical for all media. No additional fertilization was added.

The experiment was finished 7 months after planting (from 31 March 2012 to 31 October 2012), when the transplants had grown to commercial size. All of the plants were harvested, and their growth parameters and nutrient contents were assessed as described in section 2.4.

### Physical and Chemical Characteristics of the Growth Media

Physical and chemical characteristics of the seven media except particle-size distribution were determined before planting for three replicate samples (200 g per sample) per medium. Each sample was divided into two parts: one part was air-dried (3–5% moisture content), and the other was oven-dried at 65°C. When dry, the samples were crushed in a small grinder, passed through soil sieves (0.25 and 0.1 mm), sealed in plastic containers, and stored at 4°C.

Air-dried samples were used for determination of pH, electrical conductivity (EC), total organic carbon (TOC), total Kjeldahl nitrogen (TN), and total phosphorus (TP). Oven-dried samples were used for determination of total potassium (TK), Ca, Mg, and Fe contents. pH and EC were measured in a 1∶10 water soluble extract (w:v) with an MP521 pH/EC meter (Shanghai, P.R. China). TOC was determined with a ‘Liqui TOC’ total organic carbon analyzer. TN was measured by the modified micro-Kjeldahl procedure with an automatic Kjeldahl apparatus (KDY-9830, P.R. China). TP was measured by the Anti-Mo-Sb spectrophotometry method [19]. TK was determined by flame photometry [20]. Ca, Mg, and Fe were digested with sulfuric acid, and the digested liquid was analyzed by inductively coupled plasma mass spectrometry (Prodigy, America).

Bulk density (BD), water-holding capacity (WHC), total porosity (TPS), aeration porosity (AP), and water-holding porosity (WHP) were determined by the ring knife method as described by Zhang et al. [16]. A ring knife (without lid, volume of 200 cm^3^) with weight W_0_ was filled with air-dried samples (without compaction) and was weighed (W_1_). After samples were saturated by submergence in deionized water for 24 h, they were weighed again (W_2_). After that, the ring knife was covered with gauze. Then, the ring knife with saturated growth media was placed upside down on a screen until the water stopped dripping from the bottom for about 4 h. The gauze was removed, and the ring knife was then weighed again (W_3_). Finally, the ring knife with growth media was oven-dried at 65°C to constant weight (W_4_). The physical characteristics of the growth media were calculated with the following formulas:
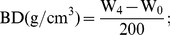


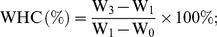


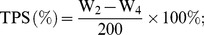


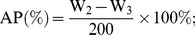


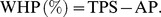



Particle-size distribution was determined as described by Gabhane et al.; air-dried samples were shaken on soil sieves with openings of 0.1, 0.25, 0.5, 1.0, and 2.0 mm (3 min of shaking on each sieve size), and the material retained on each soil sieve was weighed [21]. Whereas all other characteristics of the growth media were measured only before planting for three replicate samples (200 g per sample) per medium, particle-size distribution was determined at the start and end of the experiment for 30 pots per medium in order to observe the difference between the particle-size distribution of seven media before and after planting.

### Plant Growth Parameters at the End of the Experiment

Growth parameters were determined for 30 plants per medium. Plant height was measured with a measuring tape from media surface to the tops of the three highest leaves in each plant of each medium, and then the average was calculated [22]. Crown width was measured twice with a measuring tape in each plant (the second measurement was at a right angle to the first), and the results were averaged. Leaf number was determined by counting the number of true leaves per plant. Then, shoots and roots were washed thoroughly with deionized water, and fresh weights were determined. Roots were scanned to analyze the root morphology (total root length, total root surface area, average root diameter, total root volume, and total number of root tips) using the WinRHIZO image analysis software (Regent Instruments, WinRHIZO-EC, Canada). The length of the longest root was measured with a measuring tape. After that, shoots and roots of the same plants were oven-dried at 90°C for 30 min and then at 65°C. When constant weights were obtained, dry weights were recorded. Finally, oven-dried shoots of plants were crushed in a small grinder and passed through a 1.0 mm soil sieve for analysis of nutrient contents. Contents of TN, TP, TK, macro-nutrients (Ca and Mg), and micro-nutrients (Fe, Cu, Mn, Zn, and B) of shoots were determined using the same methods described for analysis of the growth media [3]. B content was determined by the same method used to determine the contents of Fe, Cu, Mn, and Zn.

After the plants washed, the photosynthetic pigments (chlorophylls a, b, and carotenoids) of fresh leaves were quantified as previously described [23]. Anti-Mo-Sb spectrophotometry was used for extinction measurements of the alcohol (95%) extracts of different pigments. Measurements were performed on four randomly chosen leaves per replicate; each measurement was performed twice, and every plant grown in the seven media had the same sampling weight; the mean per replicate plant was used for statistical analysis. The relative values (SPAD values) of the total chlorophyll contents were also measured with the SPAD-YLS-1 chlorophyll meter (Shandong, P.R. China).

### Statistical Analysis

Regression analyses (with ANOVA) were performed to determine the relationships between different CGW:P ratios and the physical and chemical characteristics of the seven media and the growth parameters and nutrient contents of *Calathea insignis*. Both linear and quadratic regression models were used. All statistical analyses were performed with the SPSS16.0.

## Results and Discussion

### Characteristics of the Growth Media

#### 1. Physical Characteristics

**Figure 1:**
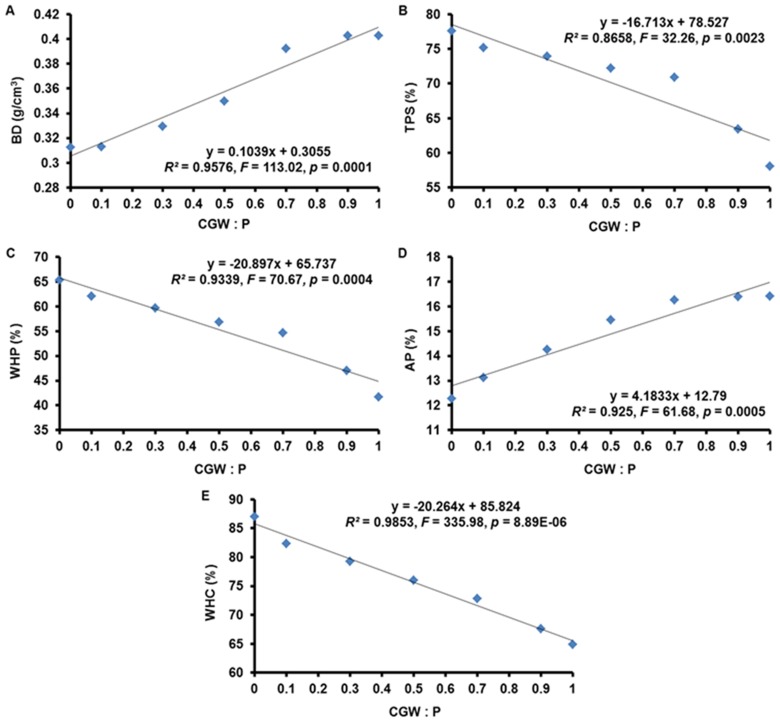
Effects of the CGW:P ratio on the physical characteristics of growth media. The linear regression equations, lines of best fit, *F* values, and adjusted *R^2^* values are shown. BD = bulk density; WHC = water-holding capacity; TPS = total porosity; AP = aeration porosity; WHP = water-holding porosity.

Linear regression demonstrated that BD highly increased (*p*<0.001) as the CGW:P ratio increased (Table 1 and Fig. 1A). The addition of sewage sludge compost and garden trimming compost also increased the BD of growth media in previous reports [4], [24]. BD values were within the acceptable range (<0.4000 g/cm^3^) in media T1 to T5 but were slightly above the acceptable range in media T6 and T7 [4].

**Table 1:** Linear regression (with ANOVA) statistics describing the effects of the CGW:P ratio on the physical characteristics of the growth media.

ANOVA table						
Characteristic	Source	Sum of Squares	df	Mean Square	*F* Value	*p*-value+
						**Prob>** ***F***
BD	Model	9.17E-03	1	9.17E-03	113.02	0.0001^***^
	*CGW:P*	*9.17E-03*	*1*	*9.17E-03*	*113.02*	*0.0001* ^***^
	*Residual*	*4.30E-04*	*5*	*8.59E-05*		
	*Corrected Total*	*0.01*	*6*			
WHC	Model	369.58	1	369.58	335.98	8.89E-06^***^
	*CGW:P*	*369.58*	*1*	*369.58*	*335.98*	*8.89E-06* ^***^
	*Residual*	*5.50*	*5*	*1.10*		
	*Corrected Total*	*375.08*	*6*			
TPS	Model	251.40	1	251.40	32.26	0.0023^**^
	*CGW:P*	*251.40*	*1*	*251.40*	*32.26*	*0.0023* ^**^
	*Residual*	*38.97*	*5*	*7.79*		
	*Corrected Total*	*290.37*	*6*			
AP	Model	15.75	1	15.75	61.68	0.0005^***^
	*CGW:P*	*15.75*	*1*	*15.75*	*61.68*	*0.0005* ^***^
	*Residual*	*1.28*	*5*	*0.26*		
	*Corrected Total*	*17.02*	*6*			
WHP	Model	393.00	1	393.00	70.67	0.0004^***^
	*CGW:P*	*393.00*	*1*	*393.00*	*70.67*	*0.0004* ^***^
	*Residual*	*27.81*	*5*	*5.56*		
	*Corrected Total*	*420.81*	*6*			

BD = bulk density; WHC = water-holding capacity; TPS = total porosity; AP = aeration porosity; WHP = water-holding porosity.

+ The *p*-value indicates the probability of a significant relationship between the physical characteristics of the growth media and the CGW:P ratio. *F* test is significant at ^n.s.^ *p*>0.05; ^***^ *p*<0.001; ^**^ *p*<0.01; ^*^ *p*<0.05. n.s.: Non-significant at *p*>0.05.

**Figure 2:**
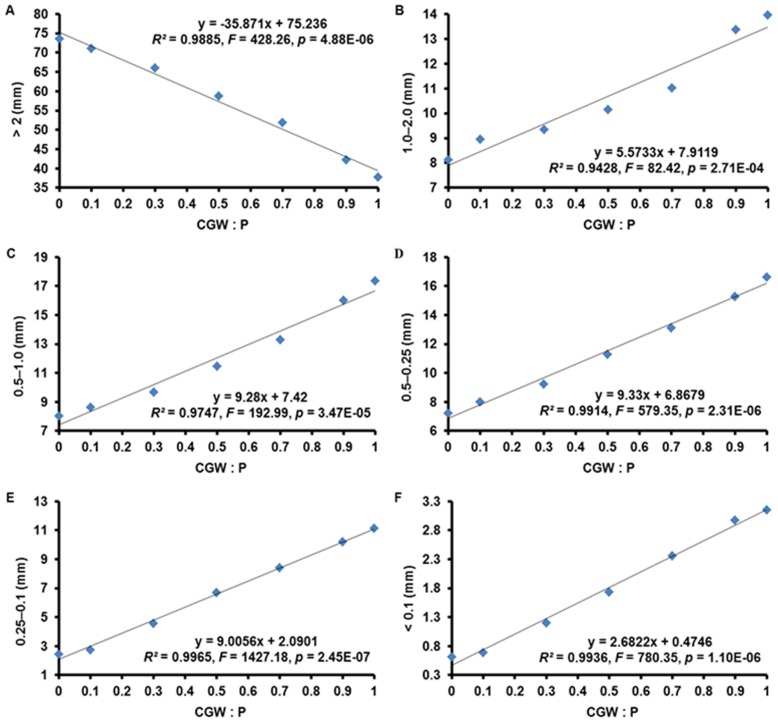
Effects of the CGW:P ratio on the particle distribution at the start of the experiment. The linear regression equations, lines of best fit, *F* values, and adjusted *R^2^* values are shown.

TPS refers to the percentage of the total volume of the substrate (without compaction) represented by pore space, and is also equivalent to the sum of AP and WHP. According to linear regression, TPS highly decreased (*p*<0.01) as the CGW:P ratio increased (Table 1 and Fig. 1B). Although TPS was highest for peat alone and was reduced by addition of CGW, TPS was within the optimal range (70.00–90.00%) for media T1 to T5. These results indicated that media T1 to T5 held sufficient water at saturation and drained slower than media T6 and T7 [25]–[27]. Similarly, WHP highly decreased (*p*<0.001) with the increasing CGW:P ratio according to linear regression analysis, which were within the acceptable range (50.00–60.00%) in media T3 to T5 but were out of that range in the other four media (Table 1 and Fig. 1C) [26], [27]. Instead, linear regression analysis showed that AP highly increased (*p*<0.001) as the CGW:P ratio increased because of the greater percentage of particles between 0.25 and 2.00 mm in CGW than in P (Table 1, Fig. 1D, and Fig. 2) [4], [25]. Media T4 to T7 had AP values characteristic of an ideal substrate (15.00–30.00%) but AP values were low in media T1 to T3; the low AP values associated with peat may limit oxygen availability and gas exchange in the growth media and thereby inhibit plant growth [26]–[28]. Addition of CGW increased air capacity while optimizing the WHC of growth media.

WHC refers to water content retained after drainage in the substrate and is expressed as a percentage of substrate weight. Like TPS, WHC highly decreased (*p*<0.001) in a linear manner as the CGW:P ratio increased, because the amount of capillary pores that retain water declined with the reduction in peat (Table 1 and Fig. 1E). WHC values for media T2 to T5 were within the ideal range (70.00–85.00%), whereas values for media T1, T6, and T7 were suboptimal [26], [27]. A reduction in WHC with addition of composted distillery waste or composted forestry waste to peat was previously reported [12], [29]. The current results were also consistent with those of Jayasinghe, who reported that WHC values decreased and AP values increased with addition of sugarcane bagasses sewage sludge compost to growth media [4].

#### 2. Chemical Characteristics

**Figure 3:**
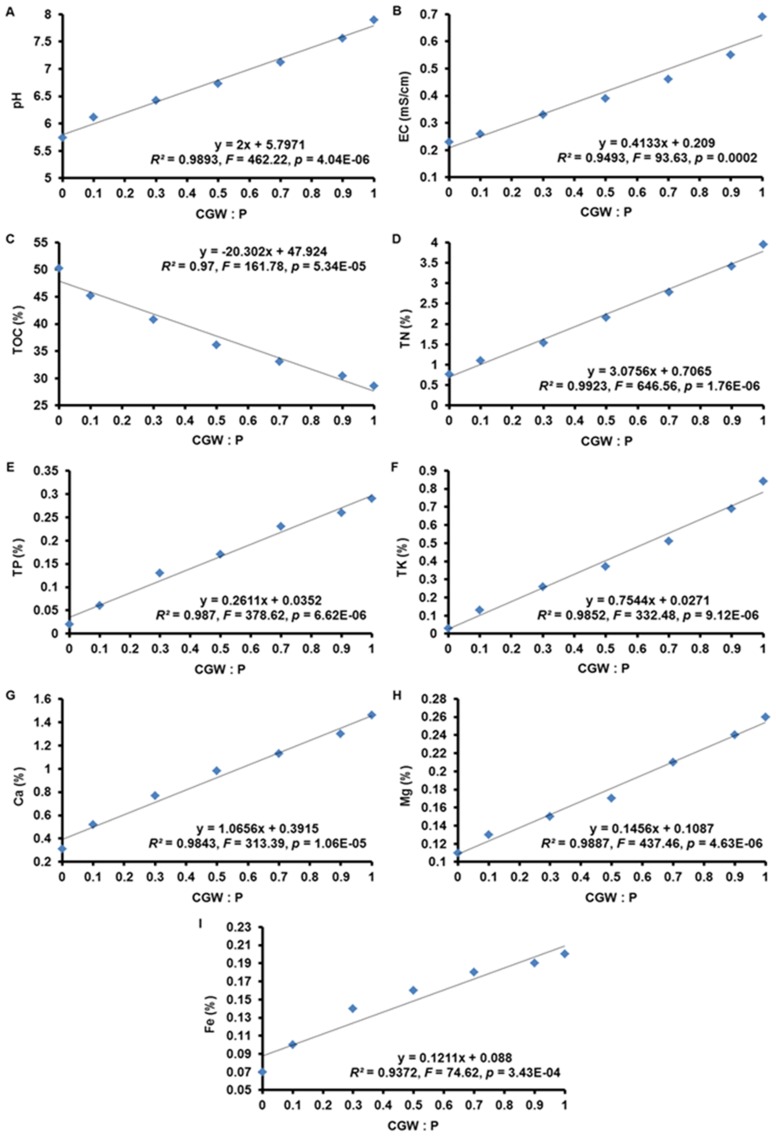
Effects of the CGW:P ratio on the chemical characteristics of growth media. The linear regression equations, lines of best fit, *F* values, and adjusted *R^2^* values are shown. EC = electrical conductivity (at 25°C); TOC = total organic carbon; TN = total Kjeldahl nitrogen; TP = total phosphorus; TK = total potassium.

Regression analyses revealed highly significant (*p*<0.001) linear relationships between the CGW:P ratio and the chemical parameters of pH, EC, TOC, TN, TP, TK, Ca, Mg, and Fe. These relationships were positive for all of these parameters except TOC (Table S1 and Fig. 3).

In general, most greenhouse-grown plants prefer a substrate with a slightly acidic pH, which increases the availability of essential nutrients [4]. The pH values significantly increased with an increase in the CGW:P ratio (Fig. 3A). The pH values of media T1 to T3 were within the established ideal range (5.2–6.5) but those in media T4 to T7 exceeded the acceptable limit [4], [26]. The high contents of alkaline elements, especially of Ca, K, and Mg, in the CGW probably increased the pH values of the mixtures.

The soluble salt level of a medium can be estimated by measuring its EC value [30]. Like pH, EC increased with addition of CGW (Fig. 3B). It was clear that the high salt contents of CGW resulted in the increased EC values in the mixtures. Previous studies with composts from garden waste and cow manure showed that salinity limited their use as a substrate component [8]. The EC values in all media except media T6 and T7, however, were below the limit for an ideal substrate (<0.5 mS/cm) suggested by de Boodt et al., and Hicklenton et al. reported that growth media could be used to cultivate plants as long as EC values were ≤1 mS/cm [27], [28]. Because EC values were less than 1 mS/cm in all media in the current study, indicating that relatively high soluble salt levels in media T6 and T7 may have little negative effects on plant growth.

As indicated by the positive slopes of the linear regressions, CGW addition increased the contents of TN, TP, TK, Ca, Mg, and Fe (Fig. 3D–Fig. 3I). These results suggested that addition of CGW increased nutrients retention and availability in the peat and could thereby increase plant growth.

That the chemical characteristics of the growth media were generally improved by addition of CGW to peat was consistent with research conducted with sugarcane bagasses sewage sludge compost and peat [4]. The chemical characteristics of peat could be optimized by addition of CGW, and this was especially evident in medium T5.

### Particle-size Distribution of the Growth Media

**Figure 4:**
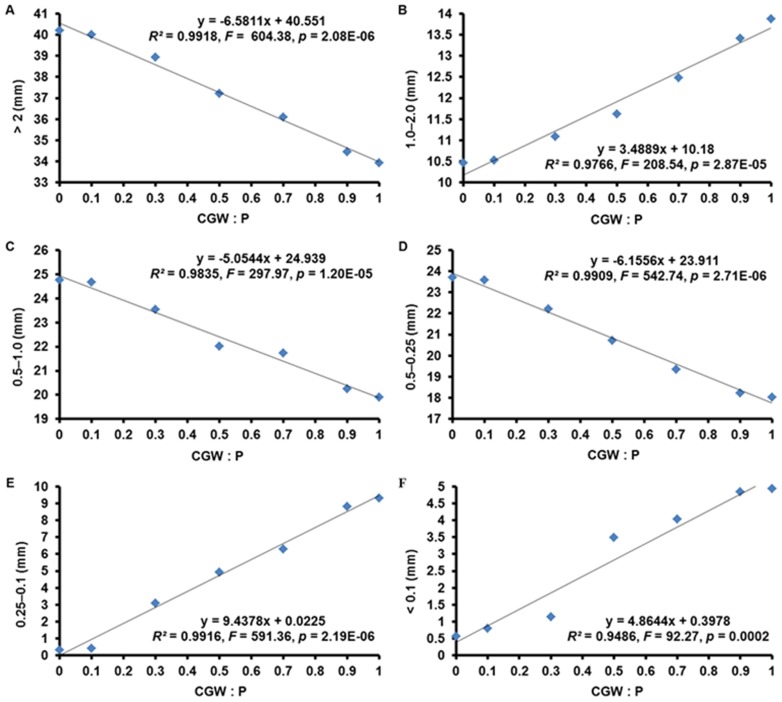
Effects of the CGW:P ratio on the particle distribution at the end of the experiment. The linear regression equations, lines of best fit, *F* values, and adjusted *R^2^* values are shown.

Regression analyses revealed linear relationships (*p*<0.001) between the particle-size distributions of the seven media and the CGW:P ratio. This was true for distributions before planting (Table 2 and Fig. 2) and after planting (Table 3 and Fig. 4).

**Table 2:** Linear regression (with ANOVA) statistics describing the effects of the CGW:P ratio on the percentage of each particle size (in mm) for each growth medium at the start of the experiment.

ANOVA table						
Particle size	Source	Sum of Squares	df	Mean Square	*F* Value	*p*-value+
						**Prob>** ***F***
>2.0	Model	1158.06	1	1158.06	428.26	4.88E-06^***^
	*CGW:P*	*1158.06*	*1*	*1158.06*	*428.26*	*4.88E-06* ^***^
	*Residual*	*13.52*	*5*	*2.70*		
	*Corrected Total*	*1171.58*	*6*			
1.0–2.0	Model	27.96	1	27.96	82.42	2.71E-04^***^
	*CGW:P*	*27.96*	*1*	*27.96*	*82.42*	*2.71E-04* ^***^
	*Residual*	*1.70*	*5*	*0.34*		
	*Corrected Total*	*29.65*	*6*			
0.5–1.0	Model	77.51	1	77.51	192.99	3.47E-05^***^
	*CGW:P*	*77.51*	*1*	*77.51*	*192.99*	*3.47E-05* ^***^
	*Residual*	*2.01*	*5*	*0.40*		
	*Corrected Total*	*79.51*	*6*			
0.5–0.25	Model	78.34	1	78.34	579.35	2.31E-06^***^
	*CGW:P*	*78.34*	*1*	*78.34*	*579.35*	*2.31E-06* ^***^
	*Residual*	*0.68*	*5*	*0.14*		
	*Corrected Total*	*79.02*	*6*			
0.25–0.1	Model	72.99	1	72.99	1427.18	2.45E-07^***^
	*CGW:P*	*72.99*	*1*	*72.99*	*1427.18*	*2.45E-07* ^***^
	*Residual*	*0.26*	*5*	*0.05*		
	*Corrected Total*	*73.25*	*6*			
<0.1	Model	6.47	1	6.47	780.35	1.10E-06^***^
	*CGW:P*	*6.47*	*1*	*6.47*	*780.35*	*1.10E-06* ^***^
	*Residual*	*0.04*	*5*	*0.01*		
	*Corrected Total*	*6.52*	*6*			

+ The *p*-value indicates the probability of a significant relationship between the percentage of each particle size (in mm) for each growth medium at the start of the experiment and the CGW:P ratio. *F* test is significant at ^n.s.^ *p*>0.05; ^***^ *p*<0.001; ^**^ *p*<0.01; ^*^ *p*<0.05. n.s.: Non-significant at *p*>0.05.

**Table 3:** Linear regression (with ANOVA) statistics describing the effects of the CGW:P ratio on the percentage of each particle size (in mm) for each growth medium at the end of the experiment.

ANOVA table						
Particle size	Source	Sum of Squares	df	Mean Square	*F* Value	*p*-value+
						**Prob>** ***F***
>2.0	Model	38.98	1	38.98	604.38	2.08E-06^***^
	*CGW:P*	*38.98*	*1*	*38.98*	*604.38*	*2.08E-06* ^***^
	*Residual*	*0.32*	*5*	*0.06*		
	*Corrected Total*	*39.30*	*6*			
1.0–2.0	Model	10.96	1	10.96	208.54	2.87E-05^***^
	*CGW:P*	*10.96*	*1*	*10.96*	*208.54*	*2.87E-05* ^***^
	*Residual*	*0.26*	*5*	*0.05*		
	*Corrected Total*	*11.22*	*6*			
0.5–1.0	Model	23.00	1	23.00	297.97	1.20E-05^***^
	*CGW:P*	*23.00*	*1*	*23.00*	*297.97*	*1.20E-05* ^***^
	*Residual*	*0.39*	*5*	*0.08*		
	*Corrected Total*	*23.38*	*6*			
0.5–0.25	Model	34.10	1	34.10	542.74	2.71E-06^***^
	*CGW:P*	*34.10*	*1*	*34.10*	*542.74*	*2.71E-06* ^***^
	*Residual*	*0.31*	*5*	*0.06*		
	*Corrected Total*	*34.42*	*6*			
0.25–0.1	Model	80.16	1	80.16	591.36	2.19E-06^***^
	*CGW:P*	*80.16*	*1*	*80.16*	*591.36*	*2.19E-06* ^***^
	*Residual*	*0.68*	*5*	*0.14*		
	*Corrected Total*	*80.84*	*6*			
<0.1	Model	21.30	1	21.30	92.27	0.0002^***^
	*CGW:P*	*21.30*	*1*	*21.30*	*92.27*	*0.0002* ^***^
	*Residual*	*1.15*	*5*	*0.23*		
	*Corrected Total*	*22.45*	*6*			

+ The *p*-value indicates the probability of a significant relationship between the percentage of each particle size (in mm) for each growth medium at the end of the experiment and the CGW:P ratio. *F* test is significant at ^n.s.^ *p*>0.05; ^***^ *p*<0.001; ^**^ *p*<0.01; ^*^ *p*<0.05. n.s.: Non-significant at *p*>0.05.

Particle-size distribution is important because it determines the balance between water and air movement in growth media. Furthermore, Benito et al. and Jayasinghe emphasized that growth media with a high percentage of particles between 0.25 and 2.00 mm are optimal for greenhouse-grown plants because they retain sufficient water, improve the availability of nutrients dissolved in water, and also provide efficient gas exchange [4], [25]. Based on linear regression, the percentage of particles between 0.25 and 2.00 mm in media T1, T2, T3, T4, T5, T6, and T7 were 23.4, 25.6, 28.2, 32.9, 37.4, 44.7, and 47.9%, respectively (Fig. 2). These results indicated that, at the start of the experiment, the percentage of particles in the optimal range of 0.25–2.00 mm of the media increased with CGW addition.

Over time, the physical structure of potting media changes because of the degradation of organic components [21]. If the growth media in the pot has experienced substantial decomposition in commercial production, additional media must be added, which obviously increases costs. Stable growth media are therefore preferable to those that degrade slowly. Tian et al. reported that growth media persistence could be determined by comparing particle-size distribution at the start and end of an incubation period [31]. According to the research method described by Tian et al., by the end of the current study, particles >1 mm decreased by 38.0, 36.8, 33.6, 29.0, 22.7, 13.8, and 7.6% in media T1, T2, T3, T4, T5, T6, and T7, respectively (Fig. 4) [31]. The results indicate that the stability of the media increased with addition of CGW.

### *Calathea insignis* Growth

**Figure 5:**
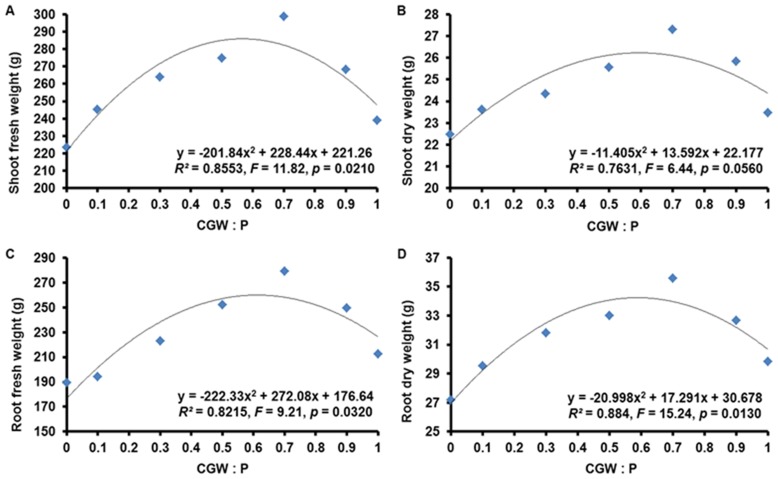
Effects of the CGW:P ratio on the biomasses of shoots and roots of *Calathea insignis*. The quadratic regression equations, lines of best fit, *F* values, and adjusted *R^2^* values are shown.

**Figure 6:**
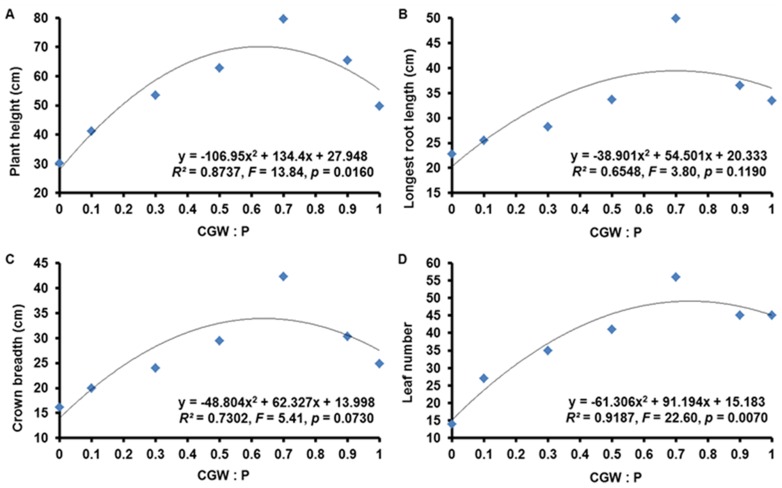
Effects of the CGW:P ratio on the growth parameters of *Calathea insignis*. The quadratic regression equations, lines of best fit, *F* values, and adjusted *R^2^* values are shown.

The plant growth parameters measured for *Calathea insignis* in the present study included fresh and dry weight of shoots and roots, plant height, the length of the longest root, crown breadth, and leaf number. Nonlinear regression analysis revealed significant (*p*<0.05) quadratic relationships between the CGW:P ratio and shoot fresh weight, root fresh weight, root dry weight, and plant height but not between the CGW:P ratio and shoot dry weight, longest root length, and crown breadth (Table 4 and Table 5; Fig. 5 and Fig. 6). Especially, leaf number response to the CGW:P ratio was highly significant (*p*<0.01).

**Table 4:** Quadratic regression (with ANOVA) statistics describing the effects of the CGW:P ratio on the biomasses (fresh and dry) of shoots and roots of *Calathea insignis*.

ANOVA table						
Biomass	Source	Sum of Squares	df	Mean Square	*F* Value	*p*-value+
						**Prob>** ***F***
Shoot fresh weight	Model	3.23E03	2	1.62*E03*	11.82	0.0210^*^
	*CGW:P*	*3.23E03*	*2*	*1.62E03*	*22.82*	*0.0210* ^*^
	*Residual*	*5.47E03*	*4*	*136.71*		
	*Corrected Total*	*3.78E03*	*6*			
Shoot dry weight	Model	12.59	2	6.30	6.44	0.0560^ n.s.^
	*CGW:P*	*12.59*	*2*	*6.30*	*6.44*	*0.0560* ^ n.s.^
	*Residual*	*3.91*	*4*	*0.98*		
	*Corrected Total*	*16.50*	*6*			
Root fresh weight	Model	5.38*E03*	2	2.69*E03*	9.21	0.0320^*^
	*CGW:P*	*5.38E03*	*2*	*2.69E03*	*9.21*	*0.0320* ^*^
	*Residual*	*1.17E03*	*4*	*2.92E03*		
	*Corrected Total*	*6.54E03*	*6*			
Root dry weight	Model	40.45	2	20.22	15.24	0.0130^*^
	*CGW:P*	*40.45*	*2*	*20.22*	*15.24*	*0.0130* ^*^
	*Residual*	*5.31*	*4*	*1.33*		
	*Corrected Total*	*45.75*	*6*			

+ The *p*-value indicates the probability of a significant relationship between the biomasses (fresh and dry) of shoots and roots of *Calathea insignis* and the CGW:P ratio. *F* test is significant at ^n.s.^ *p*>0.05; ^***^ *p*<0.001; ^**^ *p*<0.01; ^*^ *p*<0.05. n.s.: Non-significant at *p*>0.05.

**Table 5:** Quadratic regression (with ANOVA) statistics describing the effects of the CGW:P ratio on the plant height, the longest root length, crown breadth, and leaf number of *Calathea insignis*.

ANOVA table						
Parameter	Source	Sum of Squares	df	Mean Square	*F* Value	*p*-value+
						**Prob>** ***F***
Plant height	Model	1.41E03	2	703.19	13.84	0.0160^*^
	*CGW:P*	*1.41E03*	*2*	*703.19*	*13.84*	*0.0160* ^*^
	*Residual*	*203.29*	*4*	*50.82*		
	*Corrected Total*	*1.61E03*	*6*			
Longest root length	Model	315.40	2	157.70	3.80	0.1190^ n.s.^
	*CGW:P*	*315.40*	*2*	*157.70*	*3.80*	*0.1190* ^ n.s.^
	*Residual*	*166.30*	*4*	*41.58*		
	*Corrected Total*	*481.70*	*6*			
Crown breadth	Model	316.26	2	158.13	5.41	0.0730^ n.s.^
	*CGW:P*	*316.26*	*2*	*158.13*	*5.41*	*0.0730* ^ n.s.^
	*Residual*	*116.85*	*4*	*29.21*		
	*Corrected Total*	*433.11*	*6*			
Leaf number	Model	1.04E03	2	521.68	22.60	0.0070^**^
	*CGW:P*	*1.04E03*	*2*	*521.68*	*22.60*	*0.0070* ^**^
	*Residual*	*92.35*	*4*	*23.09*		
	*Corrected Total*	*1.14E03*	*6*			

+ The *p*-value indicates the probability of a significant relationship between the plant height, the longest root length, crown breadth, and leaf number of *Calathea insignis* and the CGW:P ratio. *F* test is significant at ^n.s.^ *p*>0.05; ^***^ *p*<0.001; ^**^ *p*<0.01; ^*^ *p*<0.05. n.s.: Non-significant at *p*>0.05.

After 7 months, *Calathea insignis* growth was greater in media with CGW than in peat alone. Based on quadratic regression models, addition of 0 to 70% CGW resulted in progressive increases in plant growth parameters, but parameter values began to decrease when more than 70% CGW was added (Fig. 5 and Fig. 6). Parameter values were highest in medium T5 and lowest in medium T1. Shoot dry weight, longest root length, and crown breadth showed the similar trends, though they were unrelated (*p*>0.05) to the CGW:P ratio. Sufficient nutrient supply can greatly affect plant morphology, and increasing TN, TP, TK, Ca, Mg, and Fe supply by addition of compost to the substrate enhanced both lettuce shoot and root growth in previous studies [7], [32]. As mentioned before, addition of CGW increased nutrients in the growth media, and this evidently contributed to the improved growth of *Calathea insignis* (Fig. 3). The results agree with those of Pal et al., who reported that increases in TN, TP, and TK in the growth media significantly increased the leaf number in *Polianthes tuberosa* [33].

Although addition of CGW to peat generally improved the physical and chemical characteristics of the growth media, plant growth were lower in media T6 and T7 than in medium T5. The reduced growth in media T6 and T7 relative to medium T5 may be explained by high pH and EC values, the potential presence of phytotoxic substances, and problems with the physical condition of the media with high CGW content [29], [34]. Similar results have been reported by Medina et al. and Bustamante et al. [3], [12]. Nevertheless, plant growth was generally superior in 100% CGW than in 100% peat.

### *Calathea insignis* Root Morphology

**Figure 7:**
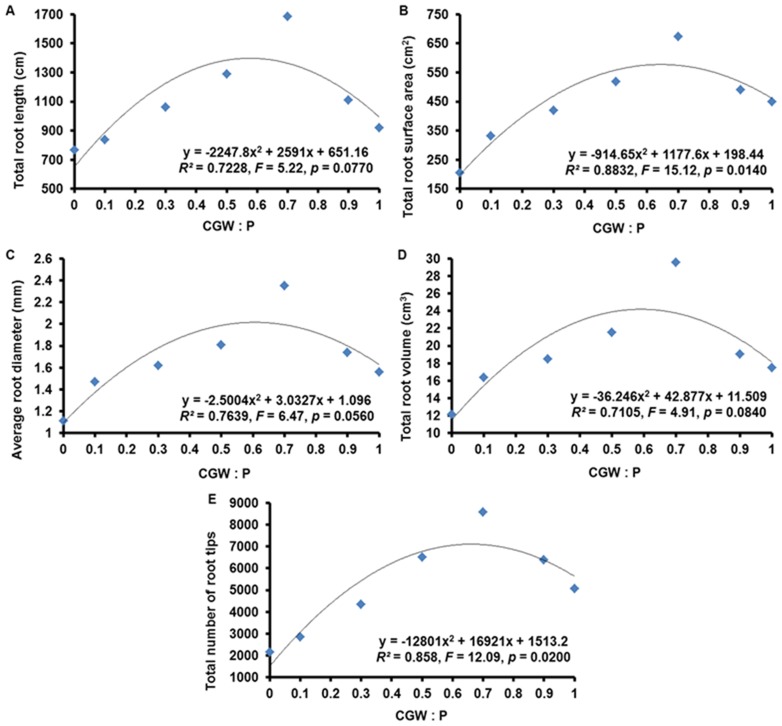
Effects of the CGW:P ratio on some characteristics of *Calathea insignis* root systems. The quadratic regression equations, lines of best fit, *F* values, and adjusted *R^2^* values are shown.

According to quadratic regression analyses, total root surface area and total number of root tips were related (*p*<0.05) to the CGW:P ratio and similar but statistically nonsignificant (*p*>0.05) trends were evident between the CGW:P ratio and total root length, average root diameter, and total root volume (Table 6 and Fig. 7).

**Table 6:** Quadratic regression (with ANOVA) statistics describing the effects of the CGW:P ratio on some characteristics of *Calathea insignis* roots.

ANOVA table						
Root characteristic	Source	Sum of Squares	df	Mean Square	*F* Value	*p*-value+
						**Prob>** ***F***
Total root length	Model	4.28E05	2	2.14E05	5.22	0.0770^ n.s.^
	*CGW:P*	*4.28E05*	*2*	*2.14E05*	*5.22*	*0.0770* ^ n.s.^
	*Residual*	*1.64E05*	*4*	*4.10E05*		
	*Corrected Total*	*5.92E05*	*6*			
Total root surface area	Model	1.16E05	2	5.78E04	15.12	0.0140^*^
	*CGW:P*	*1.16E05*	*2*	*5.78E04*	*15.12*	*0.0140* ^*^
	*Residual*	*1.53E05*	*4*	*3.82E04*		
	*Corrected Total*	*1.31E05*	*6*			
Average root diameter	Model	0.65	2	0.33	6.47	0.0560^ n.s.^
	*CGW:P*	*0.65*	*2*	*0.33*	*6.47*	*0.0560* ^ n.s.^
	*Residual*	*0.20*	*4*	*0.05*		
	*Corrected Total*	*0.86*	*6*			
Total root volume	Model	123.24	2	61.62	4.91	0.0840^ n.s.^
	*CGW:P*	*123.24*	*2*	*61.62*	*4.91*	*0.0840* ^ n.s.^
	*Residual*	*50.23*	*4*	*12.56*		
	*Corrected Total*	*173.47*	*6*			
Total number of root tips	Model	2.57E07	2	1.29E07	12.09	0.0200^*^
	*CGW:P*	*2.57E07*	*2*	*1.29E07*	*12.09*	*0.0200* ^*^
	*Residual*	*4.25E06*	*4*	*1.06E06*		
	*Corrected Total*	*3.00E07*	*6*			

+ The *p*-value indicates the probability of a significant relationship between some characteristics of *Calathea insignis* roots and the CGW:P ratio. *F* test is significant at ^n.s.^ *p*>0.05; ^***^ *p*<0.001; ^**^ *p*<0.01; ^*^ *p*<0.05. n.s.: Non-significant at *p*>0.05.

Compared to the peat alone, addition of CGW increased the root morphology parameters of *Calathea insignis*. Total root surface area and total number of root tips were highest in medium T5 and generally lowest in medium T1. Similar trends were shown for total root length, average root diameter, and total root volume, though these were unrelated (*p*>0.05) to the CGW:P ratio. As before, the increases in these root morphology parameters with addition of CGW could be attributed to the improvements in the physical and chemical characteristics of the growth media [35], [36]. However, as was the case with the other growth parameters, addition of 0 to 70% CGW resulted in progressive increases in these root morphological parameters but parameter values began to decrease when more than 70% CGW was added (Fig. 7). As before, the reductions in root morphology parameters caused by the media containing high levels of CGW might be explained by high pH and EC values, the potential presence of phytotoxic substances, and problems with physical conditions. In a previous study, high levels of forestry wastes and the solid phase of pig slurry also reduced the root growth of tomato and lettuce seedlings [29].

### Contents of Nutrients in *Calathea insignis* Shoots

**Figure 8:**
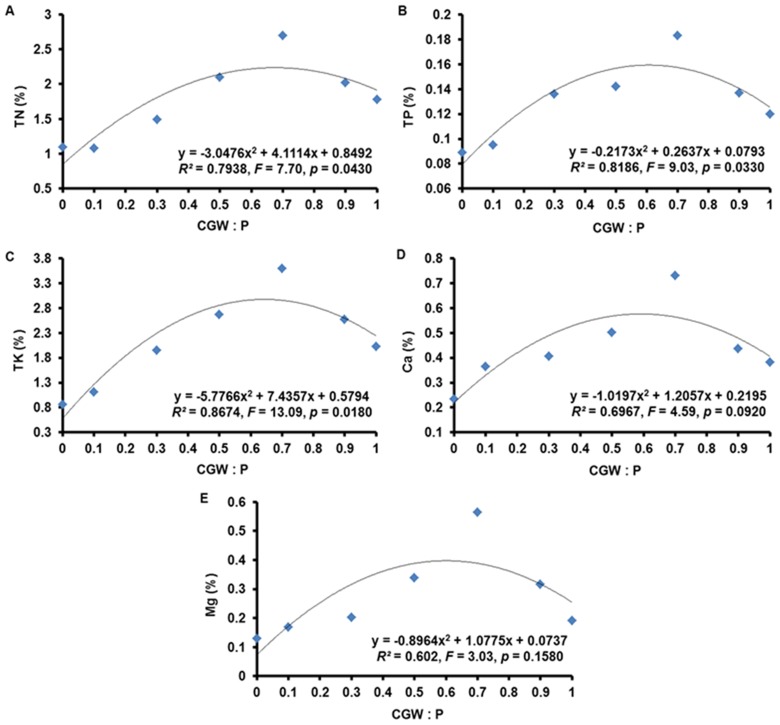
Effects of the CGW:P ratio on the contents of macro-nutrients in *Calathea insignis* leaves. The quadratic regression equations, line of best fits, *F* values, and adjusted *R^2^* values are shown.

**Figure 9:**
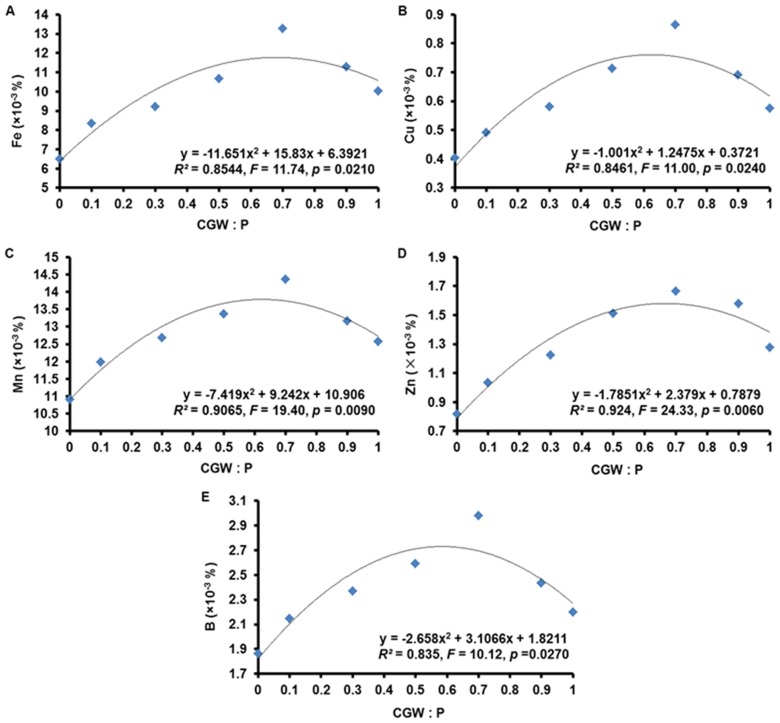
Effects of the CGW:P ratio on the contents of micro-nutrients in *Calathea insignis* leaves. The quadratic regression equations, lines of best fit, *F* values, and adjusted *R^2^* values are shown.

According to nonlinear regression analysis, the contents of TN, TP, TK, Fe, Cu, and B (but not of Ca or Mg) in *Calathea insignis* shoots were related (*p*<0.05) to the CGW:P ratio (Table 7 and Table 8; Fig. 8 and Fig. 9). Especially, response to Mn and Zn were highly significant (*p*<0.01) in the CGW:P ratio. Based on quadratic regression models, increases in CGW content from 0 to 70% resulted in progressive increases in nutrient contents but nutrient contents began to decline as the CGW level exceeded 70%. Besides, Ca and Mg showed the similar trends, though they were unrelated (*p*>0.05) to the CGW:P ratio. As with other parameters related to growth, nutrient contents were generally lowest in medium T1 and highest in medium T5, and the declines in media T6 and T7 could once again be attributed to high pH and EC values, the potential presence of phytotoxic substances, and problems with physical condition of the media containing high percentages of the CGW. In particular, high pH and excessive soluble salts (high EC values) may induce deficiencies of macro- and micro-nutrients and inhibit the ability of plant roots to absorb nutrients from the media solution [34], [37].

**Table 7:** Quadratic regression (with ANOVA) statistics describing the effects of the CGW:P ratio on the contents of macro-nutrients (TN, TP, TK, Ca, and Mg) in *Calathea insignis* leaves.

ANOVA table						
Macro-nutrient	Source	Sum of Squares	df	Mean Square	*F* Value	*p*-value+
						**Prob>** ***F***
TN	Model	1.61	2	0.81	7.70	0.0430^*^
	*CGW:P*	*1.61*	*2*	*0.81*	*7.70*	*0.0430* ^*^
	*Residual*	*0.42*	*4*	*0.11*		
	*Corrected Total*	*2.03*	*6*			
TP	Model	0.005	2	0.002	9.03	0.0330^*^
	*CGW:P*	*0.005*	*2*	*0.002*	*9.03*	*0.0330* ^*^
	*Residual*	*0.001*	*4*	*0.000*		
	*Corrected Total*	*0.006*	*6*			
TK	Model	4.60	2	2.30	13.09	0.0180^*^
	*CGW:P*	*4.60*	*2*	*2.30*	*13.09*	*0.0180* ^*^
	*Residual*	*0.70*	*4*	*0.18*		
	*Corrected Total*	*5.31*	*6*			
Ca	Model	0.10	2	0.05	4.59	0.0920^n.s.^
	*CGW:P*	*0.10*	*2*	*0.05*	*4.59*	*0.0920* ^n.s.^
	*Residual*	*0.04*	*4*	*0.01*		
	*Corrected Total*	*0.14*	*6*			
Mg	Model	0.08	2	0.04	3.03	0.1580^n.s.^
	*CGW:P*	*0.08*	*2*	*0.04*	*3.03*	*0.1580* ^n.s.^
	*Residual*	*0.05*	*4*	*0.01*		
	*Corrected Total*	*0.13*	*6*			

+ The *p*-value indicates the probability of a significant relationship between the contents of macro-nutrients (TN, TP, TK, Ca, and Mg) in *Calathea insignis* leaves and the CGW:P ratio. *F* test is significant at ^n.s.^ *p*>0.05; ^***^ *p*<0.001; ^**^ *p*<0.01; ^*^ *p*<0.05. n.s.: Non-significant at *p*>0.05.

**Table 8:** Quadratic regression (with ANOVA) statistics describing the effects of the CGW:P ratio on the contents of micro-nutrients (Fe, Cu, Mn, Zn, and B) in *Calathea insignis* leaves.

ANOVA table						
Micro-nutrient	Source	Sum of Squares	df	Mean Square	*F* Value	*p*-value+
						**Prob>** ***F***
Fe	Model	24.36	2	12.18	11.74	0.0210^*^
	*CGW:P*	*24.36*	*2*	*12.18*	*11.74*	*0.0210* ^*^
	*Residual*	*4.15*	*4*	*1.04*		
	*Corrected Total*	*28.51*	*6*			
Cu	Model	0.12	2	0.06	11.00	0.0240^*^
	*CGW:P*	*0.12*	*2*	*0.06*	*11.00*	*0.0240* ^*^
	*Residual*	*0.02*	*4*	*0.01*		
	*Corrected Total*	*0.14*	*6*			
Mn	Model	6.50	2	3.25	19.40	0.0090^**^
	*CGW:P*	*6.50*	*2*	*3.25*	*19.40*	*0.0090* ^**^
	*Residual*	*0.67*	*4*	*0.17*		
	*Corrected Total*	*7.17*	*6*			
Zn	Model	0.52	2	0.26	24.33	0.0060^**^
	*CGW:P*	*0.52*	*2*	*0.26*	*24.33*	*0.0060* ^**^
	*Residual*	*0.04*	*4*	*0.01*		
	*Corrected Total*	*0.56*	*6*			
B	Model	0.63	2	0.32	10.12	0.0270^*^
	*CGW:P*	*0.63*	*2*	*0.32*	*10.12*	*0.0270* ^*^
	*Residual*	*0.13*	*4*	*0.03*		
	*Corrected Total*	*0.76*	*6*			

+ The *p*-value indicates the probability of a significant relationship between the contents of micro-nutrients (Fe, Cu, Mn, Zn, and B) in *Calathea insignis* leaves and the CGW:P ratio. *F* test is significant at ^n.s.^ *p*>0.05; ^***^ *p*<0.001; ^**^ *p*<0.01; ^*^ *p*<0.05. n.s.: Non-significant at *p*>0.05.

### Photosynthetic Pigment Contents of *Calathea insignis* Leaves

**Figure 10:**
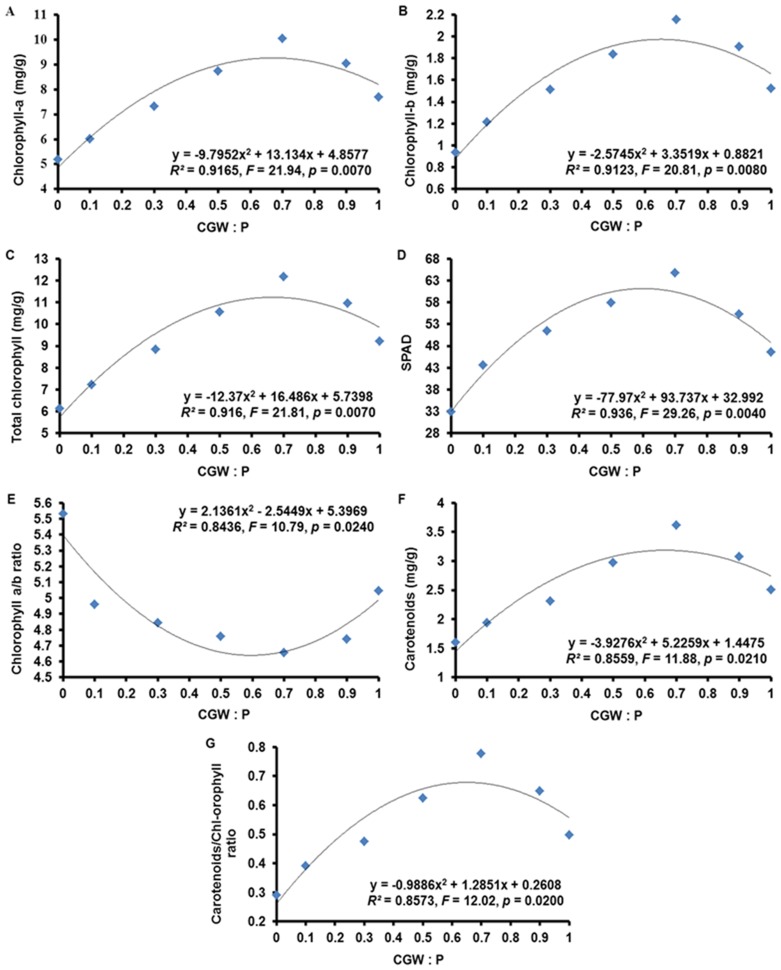
Effects of the CGW:P ratio on photosynthetic pigment contents in *Calathea insignis* leaves. The quadratic regression equations, lines of best fit, *F* values, and adjusted *R^2^* values are shown.

The pigment contents of leaves are important indicators of the status of the photosynthetic apparatus. Chlorophyll-a, chlorophyll-b, total chlorophyll, and SPAD response to the CGW:P ratio were highly significant (*p*<0.01) and similar, as indicated by nonlinear regression analysis (Table 9 and Fig. 10). Also, the response of chlorophyll a/b ratio, carotenoids, and carotenoids/chlorophyll ratio to the CGW:P ratio were significant (*p*<0.05).

**Table 9:** Quadratic regression (with ANOVA) statistics describing the effects of the CGW:P ratio on the photosynthetic pigment contents (based on fresh weight) in *Calathea insignis* leaves.

ANOVA table						
Pigment	Source	Sum of Squares	df	Mean Square	*F* Value	*p*-value+
						**Prob>** ***F***
Chlorophyll-a	Model	16.15	2	8.07	21.94	0.0070^**^
	*CGW:P*	*16.15*	*2*	*8.07*	*21.94*	*0.0070* ^**^
	*Residual*	*1.47*	*4*	*0.37*		
	*Corrected Total*	*17.62*	*6*			
Chlorophyll-b	Model	1.00	2	0.48	20.81	0.0080^**^
	*CGW:P*	*1.00*	*2*	*0.48*	*20.81*	*0.0080* ^**^
	*Residual*	*0.09*	*4*	*0.02*		
	*Corrected Total*	*1.06*	*6*			
Total chlorophyll	Model	25.00	2	12.50	21.81	0.0070^**^
	*CGW:P*	*25.00*	*2*	*12.50*	*21.81*	*0.0070* ^**^
	*Residual*	*2.29*	*4*	*0.57*		
	*Corrected Total*	*27.29*	*6*			
SPAD	Model	610.89	2	305.45	29.26	0.0040^**^
	*CGW:P*	*610.89*	*2*	*305.45*	*29.26*	*0.0040* ^**^
	*Residual*	*41.76*	*4*	*10.44*		
	*Corrected Total*	*652.66*	*6*			
Chlorophyll a/b ratio	Model	0.44	2	0.22	10.79	0.0240^*^
	*CGW:P*	*0.44*	*2*	*0.22*	*10.79*	*0.0240* ^*^
	*Residual*	*0.08*	*4*	*0.02*		
	*Corrected Total*	*0.52*	*6*			
Carotenoids	Model	2.50	2	1.25	11.88	0.0210^*^
	*CGW:P*	*2.50*	*2*	*1.25*	*11.88*	*0.0210* ^*^
	*Residual*	*0.42*	*4*	*0.11*		
	*Corrected Total*	*2.92*	*6*			
Carotenoids/Chlorophyll ratio	Model	0.14	2	0.07	12.02	0.0200^*^
	*CGW:P*	*0.14*	*2*	*0.07*	*12.02*	*0.0200* ^*^
	*Residual*	*0.02*	*4*	*0.01*		
	*Corrected Total*	*0.17*	*6*			

+ The *p*-value indicates the probability of a significant relationship between the photosynthetic pigment contents (based on fresh weight) in *Calathea insignis* leaves and the CGW:P ratio. *F* test is significant at ^n.s.^ *p*>0.05; ^***^ *p*<0.001; ^**^ *p*<0.01; ^*^ *p*<0.05. n.s.: Non-significant at *p*>0.05.

Based on quadratic regression models, increases in CGW content from 0 to 70% resulted in progressive increases in contents of chlorophyll-a, chlorophyll-b, SPAD, carotenoids, and total chlorophyll contents but these contents began to decline as CGW content exceeded 70%. These results indicate that medium T5 was the most effective at promoting the synthesis and accumulation of photosynthetic pigments; contents were lowest in medium T1. The same pattern was observed for the carotenoids/chlorophyll ratio. Opposite observation was found in the chlorophyll a/b ratio. The increases in pigment contents with CGW addition could be attributed to the nutrients added with CGW. Previous reports have documented that compost is an important source of nutrients [8], [38], [39]. N in particular is required for chlorophyll formation and photosynthesis. Mg and Fe are also required for pigment biosynthesis and are thought to be involved in chloroplast formation via protein synthesis [40], [41]. Besides, CGW could accelerate photosynthetic pigments synthesis not only by providing necessary nutrients but also by changing the physical and chemical characteristics of the growth media so as to reduce nutrients leaching and increase water-use efficiency by plants [42]. However, as mentioned above, high pH and EC values, the potential presence of phytotoxic substances, and problems with physical condition of media T6 and T7 containing high percentages of CGW may result the reduced photosynthetic pigment contents in *Calathea insignis* leaves, but the photosynthetic pigment contents of media T6 and T7 were still higher than the peat.

## Conclusions

The addition of CGW to peat improved the physical and chemical characteristics of the growth media and also reduced the rate at which the media decomposed. Plant growth was best with a medium that contained 30% P and 70% CGW. Although media with CGW contents >70% were inferior to a medium with 70% CGW content, a medium consisting of 100% CGW was still superior to a medium consisting of 100% P. Thus, CGW may be a viable alternative to peat for containerized production of *Calathea insignis*. In addition to reducing production costs in soilless culture, the use of CGW as an alternative to peat can also help protect the environment by providing an environmentally and economically benign way to dispose of green waste and by reducing the need for the mining of peat in endangered wetland ecosystems.

## Supporting Information

Table S1 **Linear regression (with ANOVA) statistics describing the effects of the CGW:P ratio on the chemical characteristics of the growth media.**
(DOC)
