# Supplementary material for: Composted Green Waste as a Substitute for Peat in Growth Media: Effects on Growth and Nutrition of Calathea insignis
Source: PLoS One. 2013 Oct 29;8(10):e78121. doi: 10.1371/journal.pone.0078121 (PMC3812227; doi:10.1371/journal.pone.0078121)
Supplement: Table S1 — Linear regression (with ANOVA) statistics describing the effects of the CGW:P ratio on the chemical characteristics of the growth media. (DOC) [file pone.0078121.s001.doc]

**Table S1.** Linear regression (with ANOVA) statistics describing the effects of the CGW:P ratio on the chemical characteristics of the growth media.

| **ANOVA table** |  |  |  |  |  |  |
| --- | --- | --- | --- | --- | --- | --- |
| **Characteristic** | **Source** | **Sum of Squares** | **df** | **Mean Square** | ***F* Value** | ***p*-value**^+^ |
|  |  |  |  |  |  | **Prob > *F*** |
| pH | Model | 3.60 | 1 | 3.60 | 462.22 | 4.04E-06^***^ |
|  | *CGW:P* | *3.60* | *1* | *3.60* | *462.22* | *4.04E-06*^***^ |
|  | *Residual* | *0.04* | *5* | *7.79E-03* |  |  |
|  | *Corrected Total* | *3.64* | *6* |  |  |  |
| EC | Model | 0.15 | 1 | 0.15 | 93.63 | 0.0002^***^ |
|  | *CGW:P* | *0.15* | *1* | *0.15* | *93.63* | *0.0002*^***^ |
|  | *Residual* | *8.21E-03* | *5* | *1.64E-03* |  |  |
|  | *Corrected Total* | *0.16* | *6* |  |  |  |
| TOC | Model | 370.96 | 1 | 370.96 | 161.78 | 5.34E-05^***^ |
|  | *CGW:P* | *370.96* | *1* | *370.96* | *161.78* | *5.34E-05*^***^ |
|  | *Residual* | *11.46* | *5* | *2.29* |  |  |
|  | *Corrected Total* | *382.43* | *6* |  |  |  |
| TN | Model | 8.51 | 1 | 8.51 | 646.56 | 1.76E-06^***^ |
|  | *CGW:P* | *8.51* | *1* | *8.51* | *646.56* | *1.76E-06*^***^ |
|  | *Residual* | *0.07* | *5* | *0.01* |  |  |
|  | *Corrected Total* | *8.58* | *6* |  |  |  |
| TP | Model | 0.06 | 1 | 0.06 | 378.62 | 6.62E-06^***^ |
|  | *CGW:P* | *0.06* | *1* | *0.06* | *378.62* | *6.62E-06*^***^ |
|  | *Residual* | *8.10E-04* | *5* | *1.62E-04* |  |  |
|  | *Corrected Total* | *0.06* | *6* |  |  |  |
| TK | Model | 0.51 | 1 | 0.51 | 332.48 | 9.12E-06^***^ |
|  | *CGW:P* | *0.51* | *1* | *0.51* | *332.48* | *9.12E-06*^***^ |
|  | *Residual* | *7.70E-03* | *5* | *1.54E-03* |  |  |
|  | *Corrected Total* | *0.52* | *6* |  |  |  |
| Ca | Model | 1.02 | 1 | 1.02 | 313.39 | 1.06E-05^***^ |
|  | *CGW:P* | *1.02* | *1* | *1.02* | *313.39* | *1.06E-05*^***^ |
|  | *Residual* | *0.02* | *5* | *3.26E-03* |  |  |
|  | *Corrected Total* | *1.04* | *6* |  |  |  |
| Mg | Model | 0.02 | 1 | 0.02 | 437.46 | 4.63E-06^***^ |
|  | *CGW:P* | *0.02* | *1* | *0.02* | *437.46* | *4.63E-06*^***^ |
|  | *Residual* | *2.18E-04* | *5* | *4.36E-05* |  |  |
|  | *Corrected Total* | *0.02* | *6* |  |  |  |
| Fe | Model | 0.01 | 1 | 0.01 | 74.62 | 3.43E-04^***^ |
|  | *CGW:P* | *0.01* | *1* | *0.01* | *74.62* | *3.43E-04*^***^ |
|  | *Residual* | *8.85E-04* | *5* | *1.77E-04* |  |  |
|  | *Corrected Total* | *0.01* | *6* |  |  |  |

EC = electrical conductivity (at 25 °C); TOC = total organic carbon; TN = total Kjeldahl nitrogen; TP = total phosphorus; TK = total potassium.

^+^ The *p*-value indicates the probability of a significant relationship between the chemical characteristics of the growth media and the CGW:P ratio. *F* test is significant at ^n.s^. *p* > 0.05; ^***^*p* < 0.001; ^**^*p* < 0.01; ^*^*p* < 0.05. n.s.: Non-significant at *p* > 0.05.
